# Cyclin B1 overexpression in conventional oral squamous cell carcinoma and verrucous carcinoma-A correlation with clinicopathological features

**DOI:** 10.4317/medoral.18220

**Published:** 2013-05-31

**Authors:** Gururaj B. Patil, Kaveri S. Hallikeri, Aswini Y. Balappanavar, Sudheer G. Hongal, P R. Sanjaya, Sheetalkumar G. Sagari

**Affiliations:** 1 BDS, MDS Reader Department of Oral and Maxillofacial Pathology. Jodhpur national University. Jodhpur dental college and Hospital. Jodhpur. Rajasthan. India; 2 BDS, MDS Professor. Department of Oral and Maxillofacial Pathology. SDM College of Dental Sciences. Dharwad. India; 3 BDS, MDS Reader. Department of Community and preventive dentistry. Jodhpur national University. Jodhpur dental college and Hospital. Jodhpur. Rajasthan. India; 4 BDS, MDS Reader. Department of Community and preventive dentistry. People dental academy. Bhopal. India; 5 BDS, MDS Reader. Department of Oral and Maxillofacial Pathology. Siddhartha Dental college and hospital. Vijayawada. India; 6 BDS, MDS Senior lecturer Department of Oral and Maxillofacial Pathology. Jodhpur national University. Jodhpur dental college and Hospital. Jodhpur. Rajasthan. India

## Abstract

Background: Nuclear localization of cyclin B1 is an indicator for cells undergoing mitotic division, and the overexpression has shown promising results as a good prognostic predictor for patients of squamous cell carcinoma (SCC). Cyclin B1 overexpression among histological grades of conventional oral squamous cell carcinoma (COSCC), as well as comparison with verrucous carcinoma (VC) has been less investigated. Study Design: Immunohistochemical expression of cyclin B1 was compared with various clinicopathological features in 30 primary COSCC and 31 primary VC cases. Result: Cyclin B1 showed significant overexpression for some clinical features for both the variants of oral squamous cell carcinoma. In histopathological variants, statistical significance was observed among grades of COSCC, as well as COSCC and its grades with VC. The concomitant increase in cyclin B1 overexpression from VC to grades COSCC was observed. Conclusion: Our study findings draw attention to cyclin B1 overexpression is involved in early carcinogenesis, cell differentiation and tumor proliferation.

** Key words:**Cyclin B1, oral squamous cell carcinoma, verrucous carcinoma, head and neck cancer.

## Introduction

Dysregulation of the cell cycle machinery is a fundamental hallmark of cancer progression and the cell programmers of proliferation, differentiation, senescence and apoptosis are intimately linked to the cell cycle regulatory machinery (1-3). Cyclin B1 is a key factor for G2-M phase transition as well as cyclin B1/Cdk complex pushes cell from G2 phase to M phase and hence this is well-known as maturation promoting factor (MPF) (4).

 This complex performs chromatin condensation, nuclear envelope breakdown, fragmentation of golgi apparatus and endoplasmic reticulum as well as spindle formation by microtubule instability. Subsequently at prophase and at beginning of anaphase an ubiquitin ligase (E3) known as the anaphase-promoting complex/cyclosome (APC/C) will get attached to cyclin B1 and Cdk complex which triggers the destruction of the mitotic cyclins (5).

The conventional tumor and several histological subtypes of squamous cell carcinoma present axiomatic morphologic features and behavior; this can be associated with differences in prognosis when they occur in the oral mucosa (6,7). Verrucous carcinoma is an distinct variant of squamous cell carcinoma, clinically characterized by an exophytic, warty, slow growing neoplasm with histologicaly as an extremely well-differentiated squamous cell carcinoma with pushing margins and non-metastasizing (6,8).

The present study is planned to explore the importance of nuclear expression of cyclin B1 in metastasizing conventional SCC, that is well differentiated squamous cell carcinoma (WDSCC), moderately differentiated squamous cell carcinoma (MDSCC) and poorly differentiated squamous cell carcinoma (PDSCC) which have not been well-studied and also to study and compare with non-metastasizing variants of oral squamous cell carcinoma that is verrucous carcinoma. Furthermore, the present study also refers to the biological behavior of tumor from the standpoint of the difference in staining pattern and overexpression of cyclin B1 in different histological grades of COSCC versus VC.

## Methodo

In this retrospective, cross-sectional study, randomly selected 30 cases of primary COSCC and 31 cases of primary VC were selected. Patients, who did not receive any kind of preoperative therapy, underwent radicular neck dissection as part of treatment and recurrence free for three year follow-up were included. 50 men and 11 women (median age 51 years) suffering from primary oral squamous cell carcinoma were selected as per pTNM stages I-III as per American join committee on cancer guidelines. Due to small sample size and for clinical convenience lesion present on palate or alveolar ridge and gingiva are categorized as lesions on bound down mucosa and loose mucosa when lesions were present on buccal mucosa or tongue.

Two pathologists decided the tumor grade and type according to the histological classification of oral cancer by the World health organization-histological malignancy grading. Histological subtypes included 30 cases of conventional oral squamous cell carcinoma, among which 11 were WDSCC, 10 MDSCC and 9 PDSCC. There were 31 cases of VC. Normal mucosa of five patients was taken as control.

-Immunohistochemistry:

Paraffin-embedded tissue sections at 4 micron thick of two to three serial sections from all 61 tumors were taken on silinated slides (Sigma Aldrich Comp. USA). All the slides were then deparaffinized through a series of xylene baths and were rehydrated in graded alcohols. Then sections were heated in a pressure cooker in 10 mM citrate buffer (pH 6.0) for 8 minutes for antigen retrieval followed by incubating in 0.3% hydrogen peroxide for 20 min to block endogenous peroxides activity. Later sections were incubated with primary anti– cyclin B1 monoclonal antibody (monoclonal, clone V152; Dako Corp, Denmark) diluted to the ration of 1:200 in tris buffered solution antibody diluent solution and incubated at room temperature for overnight in a humidifying chamber. After further incubations with secondary Antibody (45 minutes) and streptavidin peroxidase (30 minutes), visualization was performed using freshly prepared DAB (di-amino-benzidine) chromogen for 10 minutes. The slides were then counterstained with the Harris hematoxylin stain. Normal epithelium was served as positive control, and negative control was checked by omitting primary antibody.

All slides were analyzed by two investigators who were unaware of clinical information. The cyclin B1 labeling index was defined as the percentage of tumor cells displaying nuclear immunoreactivity among 1000 tumor cells, and it was calculated by counting the number of cyclin B1-stained tumor cells from appropriate areas of each tissue section. Tumors showing ≥ 5% tumor cells showing nuclear expression are considered for cyclin B1 overexpression. SPSS version 17 software was used for statistical analysis and a p value less than 0.05 considered for significance with a confidence of 95%.

## Results

**Figure 1:**
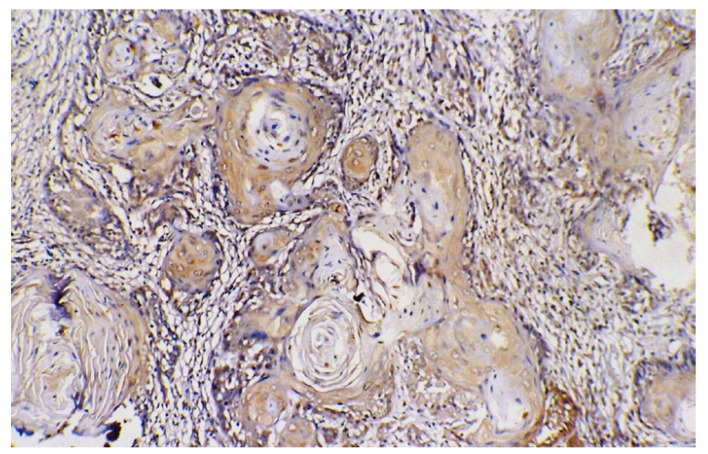
Nuclear positive cyclin B1 cells in peripheral of islands in well differentiated squamous cell carcinoma (400x).

**Figure 2:**
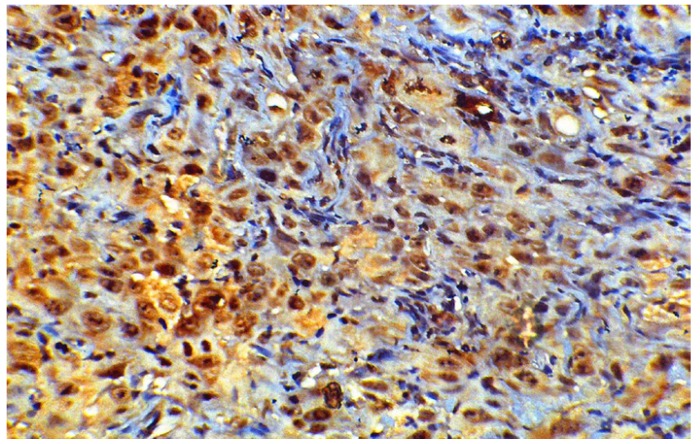
Predominant Nuclear positive cyclin B1 cells in poorly differentiated squamous cell carcinoma (400x).

**Figure 3:**
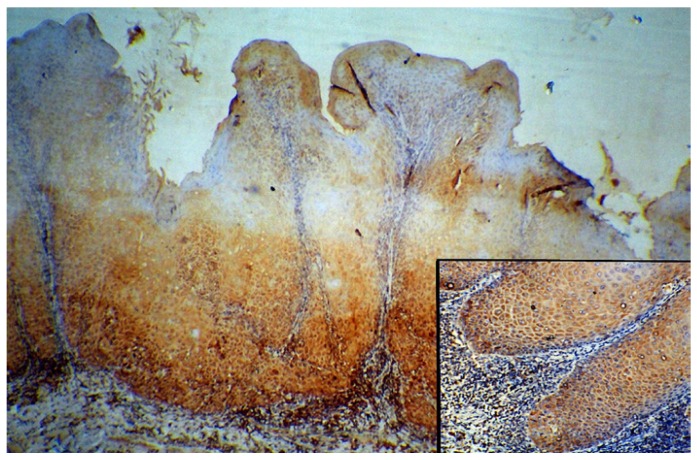
Nuclear positive cells located in basal and suprabasal cells (100x), inset showing areas of dysplastic cells in verrucous carcinoma (400x).

Cyclin B1 was expressed to some extent in all normal and cancer tissues, localized either in cytoplasm or nucleus. The WDSCC (Fig. 1) showed nuclear staining of cyclin B1 in peripheral layers of cells, in tumor islands as well as in sheets. However there were scattered areas of nuclear staining cells in MDSCC, whereas PDSCC (Fig. 2) showed predominant nuclear staining for cyclin B1. The VC (Fig. 3) has showed more uniform staining, with nuclear positive cells in basal as well as parabasal areas and superficial layer showed predominant cytoplasm staining. The dysplastic areas adjoining or overlying the lesion has showed staining with varying degree and served as internal control.

The cyclin B1 overexpression was compared for different clinical parameters for COSCC and VC ( Table 1). ANOVA and Mann Whitney U-test were used to assess the strength of association between the categories of COSCC, and VC ( Table 2).

**Table 1:**
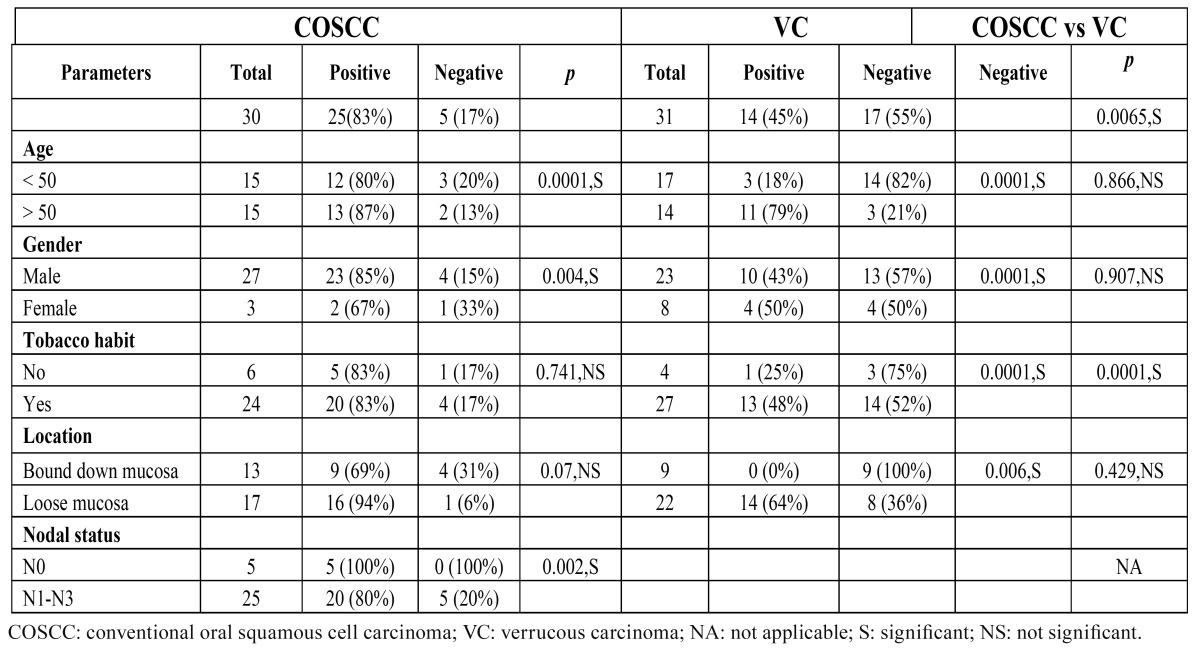
Cyclin B1 status and comparison in both variants of oral squamous cell carcinoma tumors according to clinical parameters.

**Table 2:**
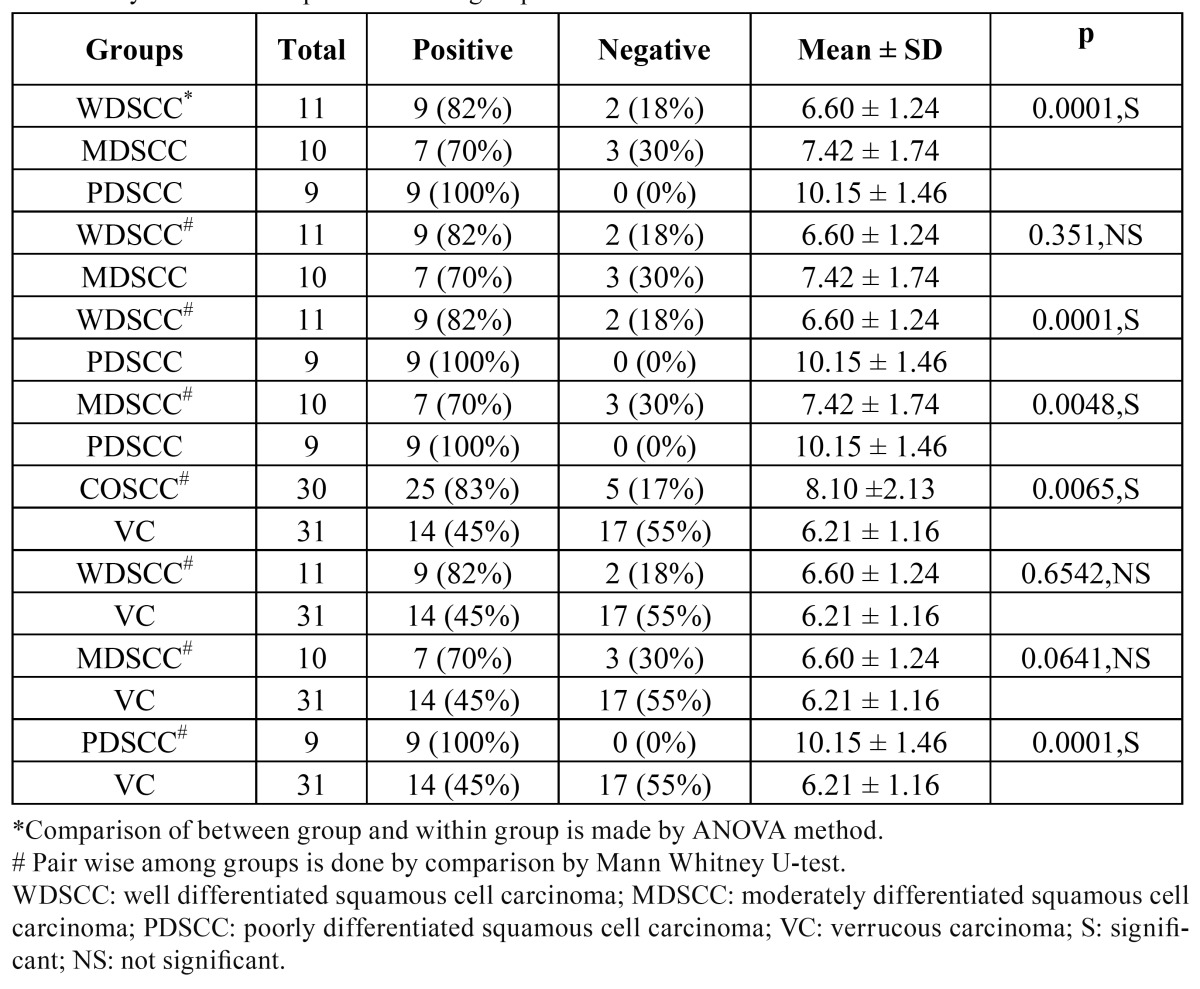
Cyclin B1 overexpression in subgroups of COSCC and VC.

## Discussion

The cell cycle markers are some of the most powerful predictors of survival for cancer patients (9-13). Most of the studies have focused on G1-S transition, but there are very few published research papers on G2-M transition using cyclin B1 among these two variants of oral carcinoma. Nuclear localization of cyclin B1 overexpression is associated with poor outcome in carcinoma of oral cavity (3,7,14,15), esophagus (10,12), breast (13), lung (16,17) and colorectal cancer (18).

In the present study, we observed that the cyclin B1 is localized either in nucleus or in cytoplasm, and was consistent with others observations (7,13,14). In breast cancer cytoplasmic expression of cyclin B1 protein was recognized in the nonmitotic phase, and nuclear expression in the mitotic phase (19). Nozoe et al. (3) concluded that the nuclear expression showed better prognostic prediction in esophageal squamous cell carcinoma than the cytoplasmic expression. Cyclin B1 was observed in 1% to 80% cells of colorectal cancer (18), Takeno et al. (9) in SCC of esophagus showed that no statistical significant difference existed in prognosis at 5% to 20% cutoffs value for overexpression.

The clinical parameters age and gender in COSCC and VC is comparable with oral carcinoma (20), and esophageal SCC (10) and in colorectal carcinoma (18). Hassan et al. (14) in SCC of tongue and Nozoe et al. (3) in esophageal SCC found contrasting results. This may be due to the difference in consideration of nuclear or cytoplasmic expression as positive for cyclin B1 overexpression. Similarly, for location of tumor the cyclin B1 overexpression in loose bound mucosa is rapidly proliferating in VC than COSCC when compared to bound down mucosa and this is in concurrence with various other sites like tongue (13-15) and esophagus carcinoma (3). Early invasion and local aggressiveness of tumor is limited by anatomical structures in VC than COSCC, contrast to this overall expression among both variants is insignificant. Tobacco habit in VC has shown very significant results, indicating tobacco has many more roles in VC initiation and local aggressiveness. The statistical insignificant correlation of overexpression in COSCC over VC is observed, this can be due to different biological role in genders and two age groups for different histological variants, than overall oral squamous cell carcinoma. This may perhaps be the change in the trend of occurrence of oral, head and neck cancer for age and gender as it noticed in younger patients (4%-6% increase) and two fold increase in females (21,22). As there is no available study in the literature, hence comparison could not be made. However it appears to be the development of carcinoma and its biological aggressive behavior is a multifactorial such as genetic susceptibility, immunological predisposition (22) allelic loss of heterozygocity (21,22). When COSCC is compared with VC, later is more common in male and also associated with tobacco habit (6,8). VC particularly associated with human papilloma virus (22) and its biological behavior is between SCC and verrucous hyperplasia (6,8) also VC has intrinsic potential for local recurrence (6).

The lymph node metastasis in COSCC is in contrast to studies in tongue (14), lung cancer (17,18) and esophageal squamous cell carcinoma (3) but comparable with Takeno et al. (9) who observed significant difference among clinical configuration and location of tumor against nodal status in esophageal cancer. Yoshida et al. (16) found vascular invasion of tumor cell correlate with over expression of cyclin B1 in non-small-cell lung cancer and Grabsch et al. (18) found no significance of cyclin B1 overexpression in colorectal cancer. The present study showed cyclin B1 is not independent of clinical parameters of COSCC and VC.

Our observation of cyclin B1 overexpression among grades of COSCC are comparable with Kushner et al. (7) report and increasing grades of dysplasia in esophagus (3,12). Hassan et al. (14) observed a trend of association of cyclin B1 and epithelial differentiation. However, correlation of cyclin B1 overexpression and lack of differentiation was observed in COSCC and histological grades by Kushner et al. (7) and Geddert et al. (12) for metaplastic, dysplastic and carcinoma sequence in Barett’s esophagus. The association between different grades of COSCC showed significant results. There appears to be cyclin B1 tend to move form cytoplasm to nucleus with grades of COSCC thus increasing the mitotic index in higher grades.

The present study showed an overlap of cyclin B1 percentage overexpression in grades of COSCC. This indicates perpetuation of cellular differentiation and may also influence the localization of cyclin B1. Our results of comparison of PDSCC, with WDSCC and MDSCC are consistent to Yoshida et al. (16) results. No statistical significant difference among WDSCC and MDSCC was observed, this concomitant inconsistency precludes use of cyclin B1 expression as an indicator for grading in contrast to Watanabe et al. (23) observation. This could be because cyclin B1 is exported to nucleus ahead of schedule by overriding nuclear export versus nuclear import, or there may be abnormal entry or exit from cell cycle than primary within cell cycle as reported by Lao-Sirieix et al. (19). This study also supports the findings of Nozoe et al. (3) that the poorly differentiated tumor has large number of cells undergoing repeated rounds of cell cycle.

Importantly the VC, a locally invasive tumor, showed a statistically significant decreased cyclin B1 overexpression, as compared with COSCC similar to de Spíndula-Filho et al. (22) study. Thus cells undergoing active mitosis and differentiation in both the carcinoma may be of different mechanism. Cyclin B1 overexpression is observed in basal and parabasal layers of VC and peripheral layers of islands of WDSCC, compared to invasive tumor front of oral squamous cell carcinoma by Watanabe et al. (23) and esophageal carcinoma by Song et al. (10) Interestingly, VC had not showed any statistical significant difference with WDSCC and MDSCC. There must be more biological significance of cyclin B1 than just proliferation in squamous cell carcinoma (3,10,11,22,24). The present study showed that the cells in carcinoma with aberrant nuclear localization of cyclin B1 not only pushes on cell from G2-M mitosis phase but also demonstrate lack of cell differentiation into next stratum.

## Conclusion

Our study findings draw attention to cyclin B1 overexpressions involvement in early carcinogenesis, cell differentiation and tumor proliferation. Clinicopathological factors have certain influence on overexpression of cyclin B1 in both the variants of SCC, as cyclin B1 localization tends to move about from cytoplasm to nucleus with lack of cell differentiation in COSCC. However overlap of nuclear overexpression among grades precludes use of cyclin B1 over expression as an indicator for grading and also cyclin B1 is not a good indicator to assess the variants of oral squamous cell carcinoma.
